# Nomogram for predicting the severity of high-risk plaques in acute coronary syndrome

**DOI:** 10.3389/fcvm.2025.1618038

**Published:** 2025-06-25

**Authors:** Miao-Na Bai, Ji-Xiang Wang, Xiao-Wei Li, Jing-Xian Wang, Yu-Hang Wang, Yin Liu, Jing Gao

**Affiliations:** ^1^ Graduate School, Clinical School of Thoracic, Tianjin Medical University, Tianjin, China; ^2^ Thoracic Clinical College, Tianjin Medical University, Tianjin, China; ^3^ Department of Cardiology, Tianjin Chest Hospital, Tianjin, China; ^4^ Cardiovascular Institute, Tianjin Chest Hospital, Tianjin, China; ^5^ Chest Hospital, Tianjin University, Tianjin, China

**Keywords:** acute coronary syndrome, nomogram, high-risk plaque, optical coherence tomography, LASSO regression algorithm

## Abstract

**Background:**

The CLIMA study [Relationship between Optical Coherence Tomography (OCT) Coronary Plaque Morphology and Clinical Outcome; NCT02883088] introduced the concept of high-risk plaque (HRP) and demonstrated that HRP was associated with a high risk of major coronary events. HRP is defined by four simultaneous characteristics: minimum lumen area (MLA) <3.5 mm^2^, fibrous cap thickness (FCT) <75 μm, lipid arc circumferential extension >180°, and macrophage infiltration. Early prediction of HRP formation is critical for preventing and treating acute coronary syndrome (ACS), but no studies have been conducted on this topic.

**Purpose:**

To identify the risk factors associated with OCT HRP in ACS and develop a risk prediction model for HRPs in ACS.

**Methods:**

A prospective observational study was conducted on patients with ACS between September 2019 and August 2022. A total of 169 patients were divided into two groups: OCT HRP (*n* = 55) and OCT non-HRP (*n* = 114) groups. Clinical data, laboratory results, and OCT characteristics of the patients were collected. Least absolute shrinkage and selection operator (LASSO) regression was used to screen variables, while multivariate logistic regression was used to create a risk prediction model. A nomogram was created, and the receiver operating characteristic curve was used to assess the model's discrimination, as well as the bootstrap method to internally validate it.

**Results:**

The most commonly observed HRP characteristic was lipid plague >180° (147 patients), followed by MLA < 3.5 mm^2^ (141 patients), macrophages (127 patients), and FCT < 75 μm (64 patients). The LASSO regression model was used to screen variables and develop an HRP risk factor model. The nomogram includes five predictors: age, BMI ≥ 25 kg/m^2^, triglycerides, low-density lipoprotein cholesterol, and Log N-terminal brain natriuretic peptide precursor. The model is highly differentiated (area under the curve 0.780, 95% confidence interval 0.705–855) and calibrated. The calibration curve and decision curve analysis demonstrated the model's clinical usefulness.

**Conclusion:**

A simple and practical nomogram for predicting HRPs accurately in patients with ACS was developed and validated, and is expected to help clinicians diagnose and prevent plaque stability.

## Introduction

Cardiovascular disease is the leading cause of death worldwide. As the unstable and progressive stage of coronary heart disease, acute coronary syndrome (ACS) is characterized by three serious and potentially fatal clinical manifestations: ST-segment elevation myocardial infarction (STEMI), non-STEMI, and unstable angina pectoris (1, 2).

In recent years, consensus has emerged that coronary atherosclerotic plaques with a propensity for thrombosis and a higher likelihood of rapid progression are commonly known as vulnerable or high-risk plaques (HRPs) (3, 4). The CLIMA study [Relationship between Optical Coherence Tomography (OCT) Coronary Plaque Morphology and Clinical Outcome; NCT02883088] introduced the concept of HRP and found that HRPs are associated with a higher risk of major coronary events (5). OCT, a high-resolution intravascular imaging technique, allows for precise identification of coronary plaque characteristics. HRPs are defined by four simultaneous characteristics: minimum lumen area (MLA) <3.5 mm^2^, fibrous cap thickness (FCT) <75 μm, lipid arc circumferential extension >180°, and macrophage infiltration (5). Previous research has found that HRPs are associated with an increased risk of cardiovascular events (6–8). Wang Ying et al. identified 274 patients with acute myocardial infarction using OCT-defined HRP plaques and followed them up for 2.2 years, finding that patients with HRP were 2.05 times more likely to have major adverse cardiovascular events than those without HRPs (7). Early prediction of HRPs formation and appropriate intervention are critical for the prevention and treatment of ACS, but no studies have been conducted on this topic.

Therefore, by analysing clinical data in conjunction with blood coherence indicators of circulation, relevant risk factors were identified, and a rapid early prediction model for HRPs was developed, providing novel insights into the prevention and treatment of ACS diseases.

## Methods

### Study population

**Figure 1:**
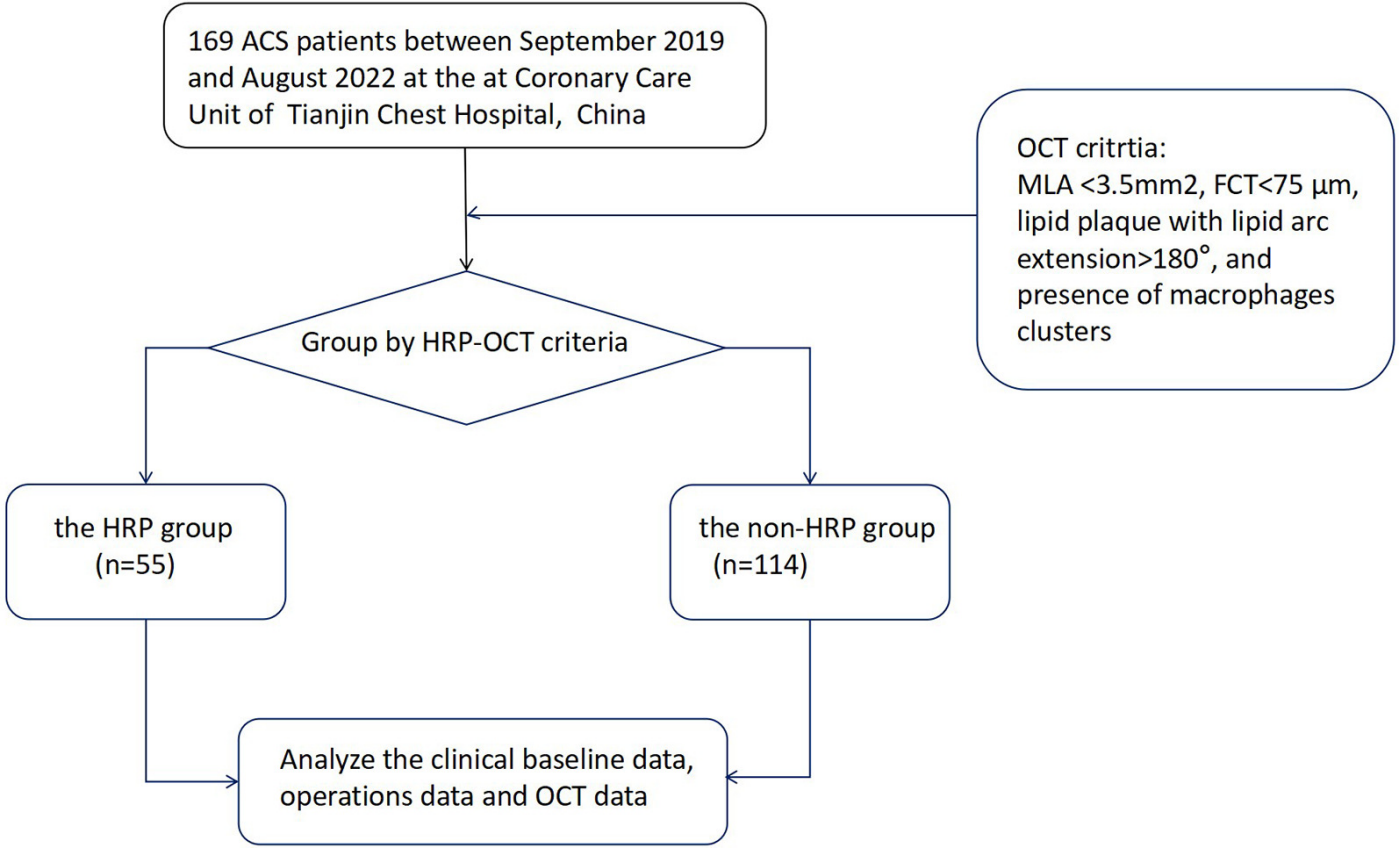
A flow diagram of the data selection process. ACS, acute coronary syndrome; OCT, optical coherence tomography; HRP, OCT-detected high-risk plaques.

This was a prospective observational study of ACS patients who underwent coronary angiography with OCT guidance between September 2019 and August 2022 at the Coronary Care Unit of Tianjin Chest Hospital, Tianjin, China. Patients aged 18 years or older with ACS who underwent coronary angiography (CAG) and pre-procedure OCT examination of the culprit lesion were enrolled (Figure 1). ACS patients were eligible if they had (1) angiographic evidence of ≥50% stenosis in ≥1 coronary vessel; (2) ischemic chest discomfort that increased or occurred at rest, and/or (3) electrocardiography (ECG) or cardiac biomarker criteria consistent with ACS. Participants with a history of chronic renal failure [glomerular filtration rate (eGFR) <60 ml/min/1.73 m^2^], sepsis, severe chronic liver disease, prior coronary stenting, or coronary artery bypass grafting were excluded from the study. The patients underwent a detailed history, full clinical examination, 12-lead ECG, echocardiography, and laboratory investigations such as the complete blood count, liver and kidney function, cardiac enzymes (troponin and creatine kinase isoenzyme MB), blood glucose, and serum lipid levels at the time of admission. All of the blood samples were assessed in the Department of Laboratory Medicine, Tianjin Chest Hospital. The concentrations of lipoprotein markers, such as low-density lipoprotein cholesterol (LDL-C), high-density lipoprotein cholesterol (HDL-C), total cholesterol (TC), and triglycerides (TG), were determined using electro-chemiluminescence immunoassay (Roche Diagnostics, Indianapolis, IN). Other laboratory parameters were measured using standard test protocols.

The study protocol followed the ethical guidelines of the 1975 Declaration of Helsinki and was approved by the Ethics Committee of Tianjin Chest Hospital (No. 2018KY-010-01). Informed consent was provided by all participants at our institution.

### Angiographic procedure

Coronary angiography was performed using a transradial or transfemoral approach with a 6F or &7F sheath. Intravascular infusion of 30–50 IU/kg unfractionated heparin was administered prior to CAG. The culprit's vessel was identified through an analysis of angiography, echocardiography, and electrocardiographic changes (ischemic ST-segment changes, T-wave inversions, and/or pathological Q wave).

### OCT imaging acquisition and definition

**Figure 2:**
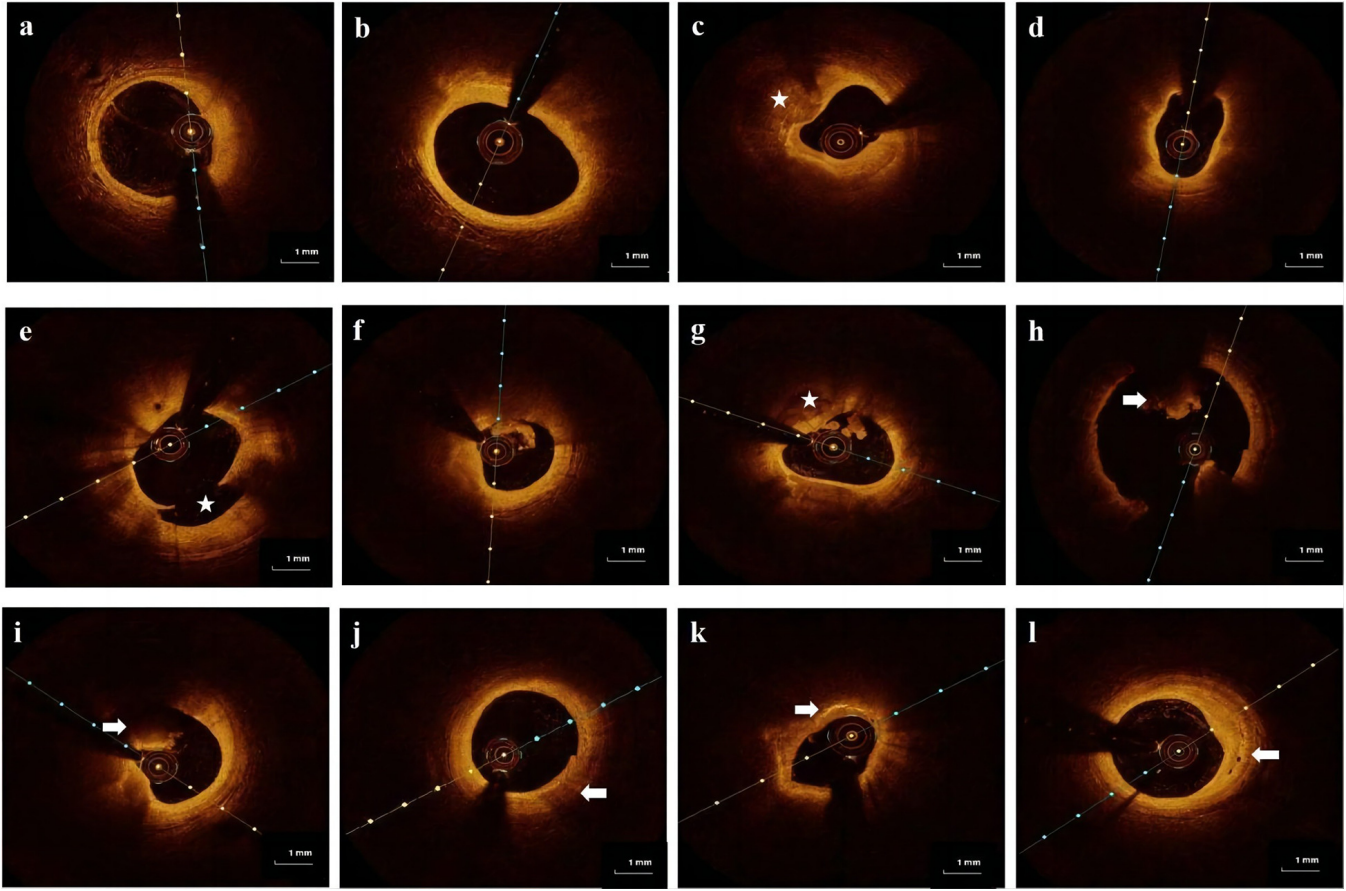
Representative cross-sectional optical coherence tomography images of the culprit's vessels: **(a)** fibrous plaque was identified as a homogeneous region with high backscatter. **(b)** Lipid-rich plaque was identified as a low signal region with a diffused border. **(c)** Calcification was detected as sharply defined, low backscattering heterogeneous regions (star). **(d)** Thin-cap fibroatheroma (TCFA) is a lipid-rich plaque with a fibrous cap thinner than 65 μm. **(e)** Plaque rupture is defined as a disruption of the fibrous cap with obvious cavity formation (star). **(f)** Plaque erosion is defined as the presence of an attached thrombus overlying an intact, visible plaque. **(g)** Calcified nodule identified as a nodular calcification protruding into the lumen and forming a thrombus (star). **(h,i)** Thrombus is defined as an irregular mass that adheres to the luminal surface, which can be a white thrombus, red thrombus (arrow), or mixed thrombus (arrow). **(j)** Macrophages are defined as signal-rich, distinct, or confluent punctuate regions with variable backward shadows (arrow). **(k)** Cholesterol crystals are linear, highly backscattering structures within plaques (arrow). **(l)** Micro-vessels are defined as black holes within a plaque that appear in at least three consecutive frames (arrow).

OCT images of the culprit coronary were obtained using the frequency-domain OPTIS imaging system (Abbott, St. Paul, Minnesota, USA). Following intracoronary administration of 0.2 mg nitroglycerin, an OCT imaging catheter was advanced distally to the lesion, and automated pullback began at a rate of 20 mm/s after manually flushing the guiding catheter with contrast media to create a nearly blood-free environment. The total length of the OCT pullback was 75 mm. Thrombus aspiration and/or gentle pre-dilation with a small balloon were performed for acute total occluded or severe stenosis lesions as needed to ensure that the OCT catheter passed through smoothly. The acquisition and analysis of OCT images has been described in detail (9–11). All OCT images were analyzed and scrutinized on an OCT workstation by two independent physicians who were blinded to the angiographic and clinical data. Inter-observer and intra-observer agreement for OCT-based HRP assessment was conducted, confirming excellent consistency and ensuring the reliability of image interpretation. The definition of image characteristics in OCT was primarily based on previous consensus (12). Culprit plaques were defined as ﬁbrous plaques [homogeneous, highly backscattering region (Figure 2a)], or lipid-rich plaques [low signal region with a diffuse border (Figure 2b)]. Calciﬁcation within plaques was deﬁned as the presence of well-defined, heterogeneous regions with low backscattering (Figure 2c). Thin-cap ﬁbroatheroma (TCFA) is deﬁned as a lipid-rich plaque with a maximum lipid arc greater than two quadrants and a thinnest FCT of <65 μm (Figure 2d). Plaque rupture was identified by its discontinuous ﬁbrous cap and clear cavity formation (Figure 2e). Plaque erosion was defined as the presence of an attached thrombus over an intact and visible plaque (Figure 2f). The calcified nodule was identified as a nodular calcification that protruded into the lumen, resulting in thrombus formation (Figure 2g). A thrombus was defined as an irregular mass that adhered to the luminal surface, which could be white, red, or mixed (Figures 2h,i). Macrophage inﬁltration was identiﬁed as signal-rich, highly reﬂective, punctate strip regions with backward shadowing, typically found at the boundary between the ﬁbrous cap and inner lipid core (Figure 2j). Cholesterol crystals were identified as linear, highly backscattering structures within plaques (Figure 2k). Micro-vessels were identified as black holes within a plaque that appeared in at least three consecutive frames (Figure 2l).

Quantitative OCT measurements contained the following information: the lipid arc was measured at 1-mm intervals throughout the lesion, and the largest arc was recorded; FCT was measured three times at the thinnest part of ﬁbrous cap, and the average value was recorded; and MLA was assessed along the length of the target lesion. The calcification score is calculated by measuring the maximum Angle, thickness, and length of the calcification and scoring it.

Previous research identified four criteria for HRP: MLA < 3.5 mm^2^, FCT < 75 μm, lipid plaque with arc extension >180°, and macrophage clusters. OCT-defined HRP is defined as the simultaneous presence of all four criteria (5).

### Statistical analysis

Continuous data is presented as mean ± standard deviation or median (interquartile ranges). Student's *t*-test or non-parametric test was employed for statistical comparisons. Categorical variables were reported as numbers (percentages), and group comparisons were made using the chi-square test or Fisher's exact test. An upset plot was created to show the prevalence and intersections of HRP characteristics (13). Least absolute shrinkage and selection operator (LASSO) reduces regression coefficients of some unimportant variables to zero by including a penalty term *λ* in model estimation. This achieves variable screening. It reduces the impact of multicollinearity, prevents model overfitting, and improves model generalizability. Using LASSO regression, according to ten-fold cross-validation, the candidate predictive variables were tested. The variables identified by LASSO that were clinically significant were incorporated into a multivariate logistic stepwise regression analysis to create a nomogram to predict the risk of HRP in patients with ACS. Draw the receiver operating characteristic (ROC) curve, calculate the area under the curve (AUC) as the evaluation metric of discriminant ability, and use the bootstrap method to validate the model internally. The calibration curve was used to assess the calibration force of the final model, and a decision curve analysis (DCA) was performed to ensure the model's clinical feasibility.

Analyses were performed using IBM SPSS Statistics version 25.0 (IBM SPSS Statistics, IBM Corporation, Armonk, New York) and R 4.3.1 (http://www.rproject.org/) statistical packages. A bilateral *P*-value of <0.05 was considered statistically significant.

## Results

### Baseline characteristics

Table 1 shows the baseline and angiographic characteristics. Among the 169 enrolled patients, the average age was 58 ± 12.65 years. 85.2% were males, and 52.1% had unstable angina pectoris. Patients with HRP had a higher Body Mass Index (BMI) (25.35 [23.78, 27.76] vs. 24.22 [23.12, 25.95], *P* = 0.010) than the non-HRP group. Furthermore, there was no obvious difference in the distribution of culprit vessels. The angiographic findings for the culprit vessels were also presented.

**Table 1:** Baseline characteristics of the patients.

Variables	Overall (*n* = 169)	Non-HRP (*n* = 114)	HRP (*n* = 55)	*P* value
Ages (years)	58.00 ± 12.65	57.44 ± 12.59	59.16 ± 12.83	0.408
Male, *n* (%)	144 (85.2)	98 (86.0)	46 (83.6)	0.690
BMI (kg/m^2^)	24.69 (23.13, 26.30)	24.22 (23.12, 25.95)	25.35 (23.78, 27.76)	0.010
BMI ≥ 25 (kg/m^2^)	78 (46.15)	44 (38.60)	34 (61.82)	0.005
Medical history, *n* (%)				
Hypertension	96 (56.8)	65 (57.0)	31 (56.4)	0.936
Diabetes	47 (27.8)	27 (23.7)	20 (36.4)	0.085
Previous MI	26 (15.4)	19 (16.7)	7 (12.7)	0.506
Previous stroke	15 (8.9)	10 (8.8)	5 (9.1)	0.946
Family history of CAD	9 (5.3)	5 (4.4)	4 (7.3)	0.676
Smoking	94 (55.6)	65 (57.0)	29 (52.7)	0.599
Drinking	43 (25.4)	30 (26.3)	13 (23.6)	0.708
Type of ACS, *n* (%)				0.142
STEMI	64 (37.9)	43 (37.7)	21 (38.2)	
NSTEMI	17 (10.1)	8 (7.0)	9 (16.4)	
UA	88 (52.1)	63 (55.3)	25 (45.5)	
Admission sign				
LAD (mm)	36.00 (35.00, 39.00)	36.00 (34.00, 39.00)	36.00 (35.00, 38.00)	0.487
LVED (mm)	51.00 (48.00, 54.00)	51.00 (48.00, 53.75)	52.00 (49.00, 54.00)	0.382
LVEF (%)	57.00 (50.00, 61.50)	57.00 (50.75, 62.00)	57.00 (50.00, 61.00)	0.864
PAP (mmHg)	30.00 (30.00, 30.00)	30.00 (30.00, 30.00)	30.00 (30.00, 30.00)	0.876
Laboratory data				
Blood routine				
WBC (10^9^/L)	7.39 (6.18, 9.53)	7.20 (6.13, 9.32)	7.99 (3.26, 9.74)	0.159
Neutrophil (10^9^/L)	4.90 (3.72, 7.15)	4.81 (3.77, 6.71)	5.04 (3.46, 7.79)	0.711
Lymphocyte	1.79 (1.39, 2.10)	1.79 (1.41, 2.08)	1.84 (1.33, 2.16)	0.899
Monocyte	0.46 (0.36, 0.59)	0.45 (0.36, 0.58)	0.26 (0.36, 0.64)	0.315
RBC (10^12^/L)	4.63 (4.35, 4.95)	4.62 (4.34, 4.89)	4.75 (4.42, 5.03)	0.238
Hemoglobin (g/L)	143.00 (134.00, 154.00)	144.00 (134.75, 152.00)	142.00 (131.00, 160.00)	0.760
Platelets (10^9^/L)	211.00 (184.00, 251.00)	211.00 (178.50, 246.75)	212.00 (190.00, 265.00)	0.388
Inflammation indicators				
Hs-CRP (mg/L)	1.93 (1.13, 5.16)	1.68 (0.95, 4.15)	2.98 (1.42, .45)	0.012
CLR	1.16 (0.53, 3.36)	0.97 (0.56, 2.30)	1.59 (0.87, 4.94)	0.016
Kidney function indicators				
Creatinine (μmol/L)	75.00 (67.00, 86.50)	75.00 (67.75, 87.00)	73.00 (67.00, 83.00)	0.561
Urea (mmol/L)	4.90 (3.80, 5.90)	5.00 (3.98, 6.00)	4.50 (3.70, 5.70)	0.130
Uric acid (μmol/L)	322.00 (365.00, 389.50)	318.50 (263.00, 394.00)	339.00 (268.00, 381.00)	0.876
Liver function indicators				
TBA (μmol/L)	1.83 (0.97, 3.12)	1.85 (1.02, 3.34)	1.74 (0.80, 2.58)	0.104
ALB (g/L)	42.70 (40.25, 45.00)	42.80 (40.25, 45.00)	42.30 (40.20, 45.10)	0.792
ALT (U/L)	26.40 (16.85, 42.60)	23.70 (16.00, 42.90)	32.55 (19.13, 41.78)	0.172
AST (U/L)	26.40 (18.40, 82.20)	26.20 (17.85, 71.35)	29.25 (19.15, 127.08)	0.265
LDH (U/L)	199.00 (164.50, 365.50)	188.50 (163.00, 319.50)	235.00 (172.00–610.00)	0.044
Glycolipid metabolism indicators				
Glucose (mmol/L)	5.89 (5.13, 7.12)	5.79 (4.97, 6.84)	6.18 (5.62, 8.07)	0.004
HbA1c (%)	5.90 (5.75, 6.80)	5.90 (5.60, 6.40)	6.00 (5.90, 7.00)	0.026
HCY (μmol/L)	12.53 (10.14, 19.01)	12.49 (10.25, 21.48)	12.67 (10.01, 18.00)	0.540
TG (mmol/L)	1.63 (1.28, 2.34)	1.48 (1.20, 2.15)	2.18 (1.51, 2.89)	<0.001
TC (mmol/L)	4.11 (3.50, 4.82)	4.09 (3.23, 4.65)	4.44 (3.88, 5.43)	0.001
HDL-C (mmol/L)	0.98 (0.84, 1.15)	1.00 (0.87, 1.23)	0.95 (0.80, 1.05)	0.030
LDL-C (mmol/L)	2.62 (1.93, 3.26)	2.52 (1.72, 3.11)	2.75 (2.29, 3.50)	0.003
LHR	2.73 (1.84, 3.51)	2.39 (1.71, 3.34)	3.15 (2.54, 3.69)	<0.001
TRLC (mmol/L)	0.41 (0.25, 0.61)	0.38 (0.24, 0.58)	0.47 (0.25, 0.82)	0.093
Apo B (g/L)	0.93 (0.80, 1.09)	0.90 (0.67, 1.08)	1.08 (0.90, 1.22)	0.001
Apo A1 (g/L)	1.19 (1.08, 1.30)	1.19 (1.07, 1.29)	1.19 (1.08, 1.35)	0.319
ApoB/ApoA1	0.78 (0.65, 0.97)	0.76 (0.62, 0.93)	0.91 (0.76, 1.01)	0.006
Lp(a) (mmol/L)	125.55 (72.35, 205.10)	125.55 (55.88, 193.21)	150.30 (114.40, 212.44)	0.054
Cardiac function indicators				
Log NT-proBNP (pg/ml)	2.21 (2.01, 2.53)	2.09 (1.74, 2.51)	2.50 (2.21, 2.59)	<0.001
hs-TnT (ng/ml)	0.03 (0.01, 1.17)	0.02 (0.01, 0.95)	0.24 (0.01, 3.40)	0.073
CK (U/L)	111.00 (71.50, 646.50)	103.50 (71.00, 476.75)	157.00 (74.00, 1, 283.00)	0.146
CK-MB (U/L)	17.00 (13.50, 76.00)	16.50 (13.00, 52.00)	21.00 (14.00, 95.00)	0.102
Coagulation indicators				
D-Dimer (mg/L)	0.27 (0.22, 0.38)	0.27 (0.22, 0.38)	0.28 (0.21, 0.38)	0.716
PT (sec)	12.90 (12.50, 13.45)	12.90 (12.50, 13.43)	12.90 (12.50, 13.50)	0.795
TT (sec)	17.70 (16.70, 18.60)	17.70 (16.68, 18.50)	17.60 (16.90, 18.90)	0.634
Fbg (g/L)	3.10 (2.80, 3.68)	3.12 (2.80, 3.72)	3.09 (2.78, 3.65)	0.946
CAG, *n* (%)				
Single-vessel disease	57 (33.7)	42 (36.8)	15 (27.3)	0.218
Double-vessel disease	58 (34.3)	40 (35.1)	18 (32.7)	0.762
Triple-vessel disease	52 (30.8)	31 (27.2)	21 (38.2)	0.147
Left main	31 (18.3)	22 (19.3)	9 (16.4)	0.644
Culprit vessel				0.315
LAD, *n* (%)	118 (69.8)	83 (72.8)	35 (63.6)	
LCX, *n* (%)	12 (7.1)	6 (5.3)	6 (10.9)	
RCA, *n* (%)	39 (23.1)	25 (21.9)	14 (25.5)	

Continuous data are presented as mean ± standard deviation or median (interquartile ranges). Categorical data are presented as number (%). Student's *t*-test or non-parametric test was employed for statistical comparisons. Categorical variables were reported as numbers (percentages), and group comparisons were made using the chi-square test or Fisher's exact test. BMI, body mass index; MI, myocardial infarction; CAD, coronary artery disease; ACS, acute coronary syndrome; STEMI, ST-segment elevation myocardial infarction; NSTEMI, non-ST-segment elevation myocardial infarction; UA, unstable angina; LVEF, left ventricular ejection fraction; WBC, white blood cells; RBC, red blood cells; Hs-CRP, high-sensitivity C-reactive protein; CLR, C-reactive protein to lymphocyte ratio; TBA, total bile acid; ALB, albumin; ALT, alanine aminotransferase; AST, aspartate aminotransferase; LDH, lactate dehydrogenase; HbA1c, glycated hemoglobin A 1c; HCY, homocysteine; TG, triglycerides; TC, total cholesterol; HDL-C, high-density lipoprotein cholesterol; LDL-C, low-density lipoprotein cholesterol; LHR, low-density lipoprotein cholesterol to high-density lipoprotein cholesterol ratio; TRLC, rich in triglyceride lipoprotein cholesterol; Apo B, apolipoprotein B; Apo A1, apolipoprotein A1; LP(a), lipoprotein(a); NT-proBNP, N-terminal pro-B-type natriuretic peptide; hs-TnT, high-sensitivity troponin T; CK, creatine kinase; CK-MB, creatine kinase MB; PT, prothrombin time; TT, thrombin time; Fbg, fibrinogen; LAD, left anterior descending; LCX, left circumflex artery; RCA, right coronary artery.

There were significant variations in TG (*P* < 0.001), HDL (*P* = 0.030), LDL-C (*P* = 0.003), apolipoprotein b (*P* = 0.001), high-sensitivity C-reactive protein (Hs-CRP) (*P* = 0.012), and Log N-terminal brain natriuretic peptide precursor (Log NT-proBNP) (*P* < 0.001) between the two groups. There were no significant differences between the two groups in other circulating blood-related parameters (all *P* ≥ 0.05).

### OCT findings and HRP characteristics

Table 2 displays the OCT characteristics. In addition to the OCT-defined HRP characteristics, patients with HRP had significantly higher rates of plaque rupture (36.4 vs. 18.4, *P* = 0.011), micro-vessels (72.2 vs. 48.7, *P* = 0.004), thrombus (47.3 vs. 26.3, *P* = 0.007), cholesterol crystal (87.3 vs. 57.0, *P* < 0.001), and TCFA (54.5 vs. 1.8, *P* < .001) compared to the non-HRP group. However, the difference in the prevalence of calcification between the HRP and non-HRP groups was not statistically significant.

**Table 2:** OCT analysis.

OCT analysis	Overall (*n* = 169)	Non-HRP (*n* = 114)	HRP (*n* = 55)	*P* value
Qualitative OCT analysis				
Macrophage	127 (75.1)	72 (63.2)	55 (100.0)	<0.001
Micro-vessels	94 (56.3)	55 (48.7)	39 (72.2)	0.004
Cholesterol crystal	113 (66.9)	65 (57.0)	48 (87.3)	<0.001
Plaque rupture	41 (24.3)	21 (18.4)	20 (36.4)	0.011
Plaque erosion	19 (11.2)	11 (9.6)	8 (14.5)	0.345
Calcification	4 (2.4)	2 (1.8)	2 (3.6)	0.830
Thrombus	56 (33.1)	20 (26.3)	26 (47.3)	0.007
MLA < 3.5 mm^2^	141 (83.4)	86 (75.4)	55 (100)	<0.001
FCT < 75 μm	65 (38.5)	10 (8.8)	55 (100)	<0.001
TCFA	32 (18.9)	2 (1.8)	30 (54.5)	<0.001
Quantitative OCT analysis				
Max lipid arc (degree)	360.0 (269.7, 360.0)	310.5 (206.5, 360.0)	360.0 (360.0, 360.0)	<0.001
Mean lipid arc (degree)	177.4 (138.9, 213.2)	162.3 (122.8, 198.3)	202.3 (172.6, 233.0)	<0.001
Lipid length (mm)	18.0 (12.5, 26.3)	16.0 (10.52, 22.5)	23.0 (15.4, 33.8)	<0.001
Lipid index	3,135.7 (1,805.5, 4,789.6)	2,486.9 (1,458.8, 3,960.8)	4,751.5 (2,755.0, 6,636.0)	<0.001
Calcification score	0.0 (0.0, 1.0)	0.0 (0.0, 1.3)	0.0 (0.0, 1.0)	0.295
MLA (mm^2^)	1.6 (1.2, 2.8)	1.7 (1.2, 3.2)	1.5 (1.1, 2.0)	0.046
Area stenosis (%)	74.8 (65.1, 81.0)	74.2 (64.0, 80.3)	77.3 (66.9, 81.1)	0.078
FCT (um)	90.0 (69.0, 128.5)	115.0 (89.3, 164.5)	64.0 (59.0, 73.0)	<0.001

Continuous data are presented as mean ± standard deviation or median (interquartile ranges). Categorical data are presented as number (%). Student's *t*-test or non-parametric test was employed for statistical comparisons. Categorical variables were reported as numbers (percentages), and group comparisons were made using the chi-square test or Fisher's exact test. OCT, optical coherence tomography; MLA, minimal lumen area; FCT, fibrous cap thickness; TCFA, thin-cap fibroatheroma; HRP, OCT-defined high-risk plaques.

**Figure 3:**
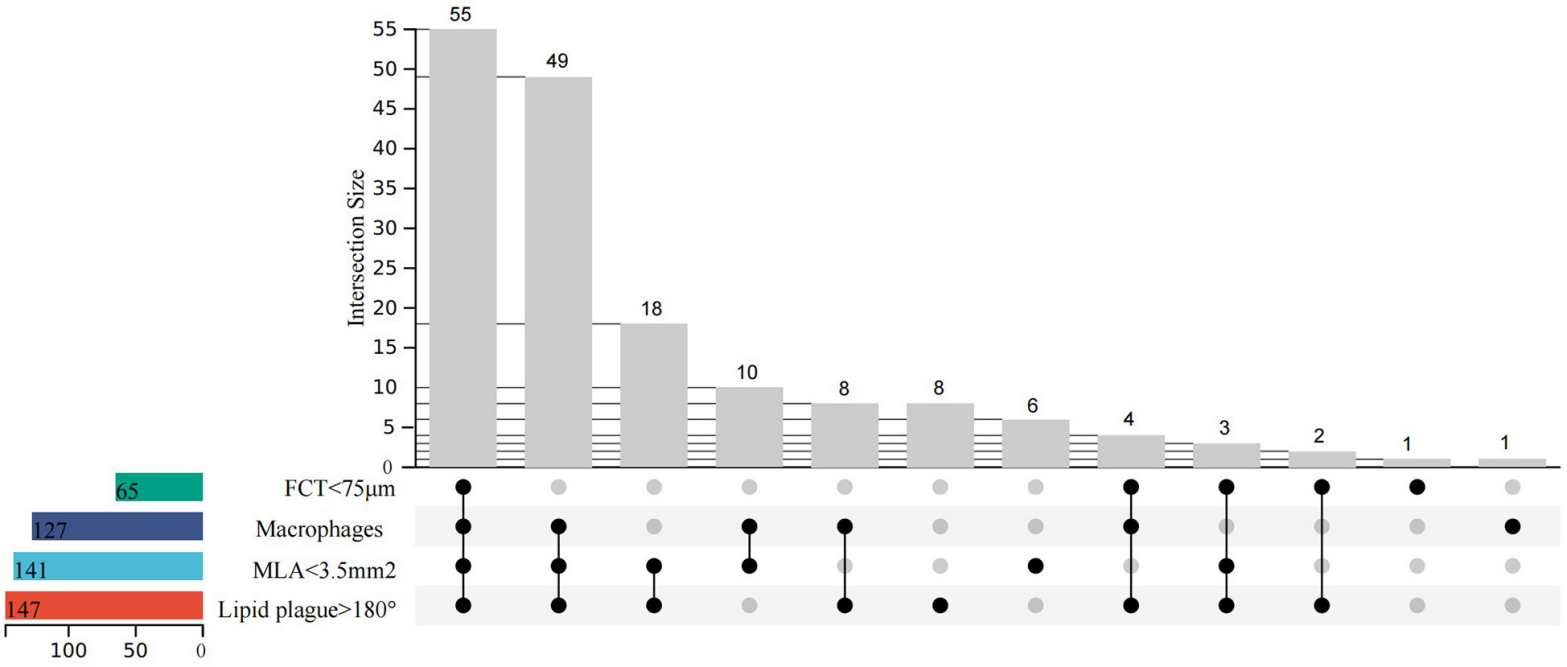
Upset plot of high-risk characteristics and combinations (minimal lumen area <3.5 mm^2^, fibrous cap thickness <75 μm, lipid arc circumferential extension >180°, and presence of macrophages). *N* = 169.

Figure 3 depicts the prevalence of individual and HRP characteristics and their intersections. The most common HRP characteristic was MLA < 3.5 mm^2^ (83.4% of enrolled patients), followed by macrophage infiltration (75.1%), FCT < 75 μm (38.5%), and TCFA (18.9%).

### Identification of predictive factors and construction of nomograms

**Figure 4:**
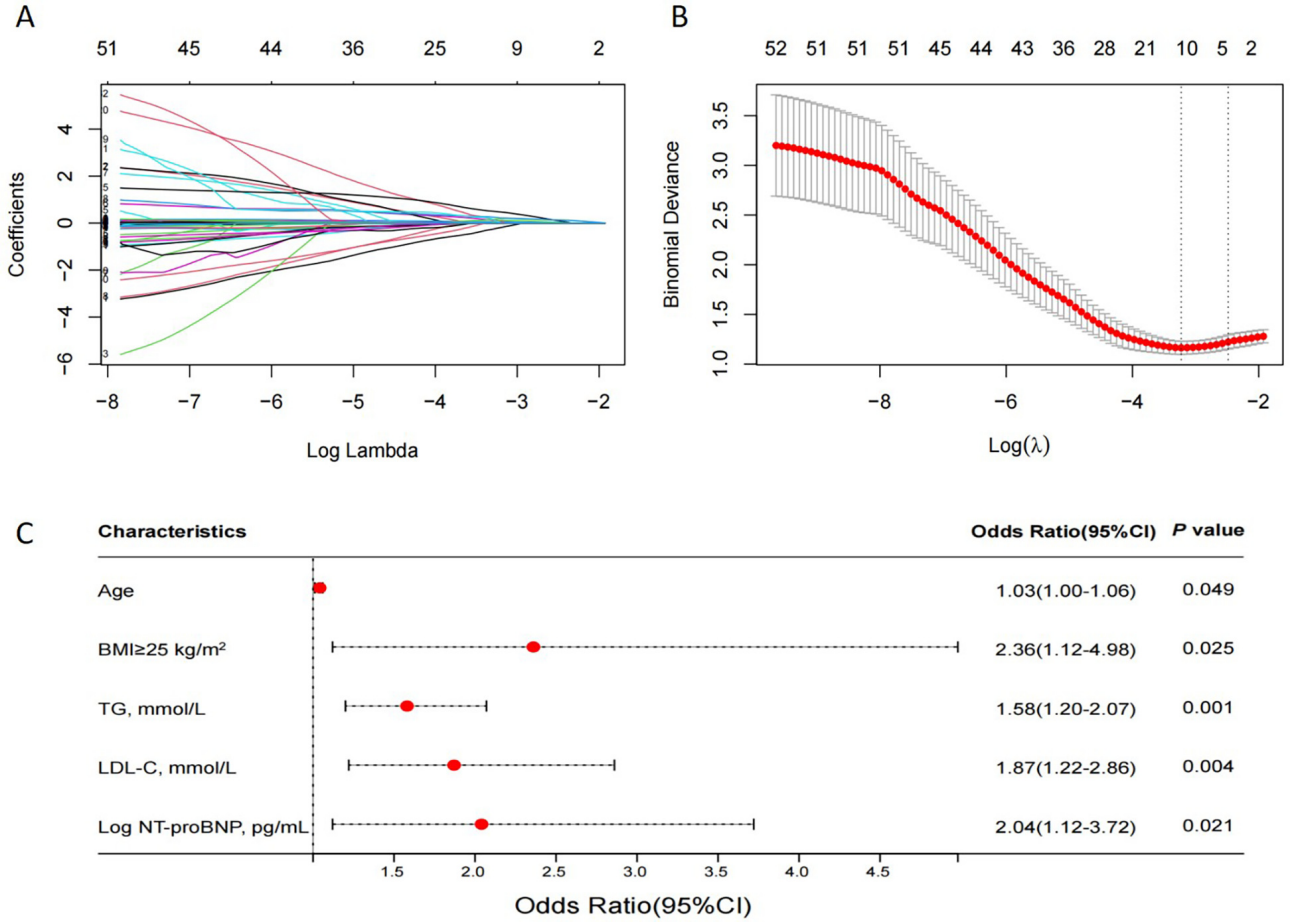
The LASSO regression model tested the factors affecting HRPs. **(A)** LASSO regression coefficient path diagram. **(B)** Cross-validation curve of LASSO regression, filtering out five predictor variables with non-zero coefficients using optimal lambda. **(C)** Multivariate logistic analysis forest plot of predictors. LASSO, least absolute shrinkage and selection operator; HRP, OCT-defined high-risk plaques; *N* = 169.

**Figure 5:**
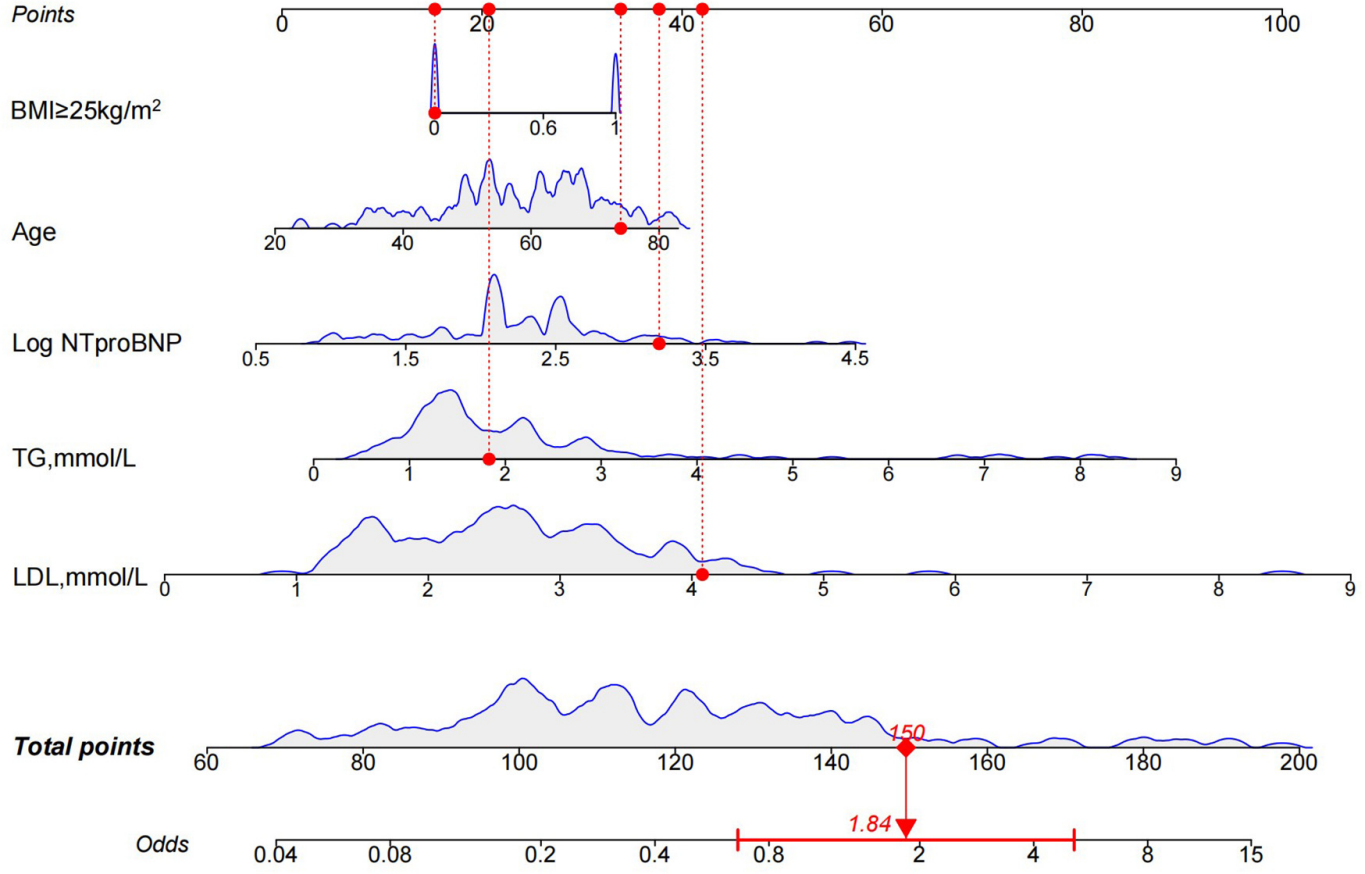
Nomogram for the HRP risk factor model. The nomogram is constructed based on age, BMI ≥ 25 kg/m^2^, TG, LDL-C, and Log NT-proBNP to assign the probability of high-risk plaques. HRP, high-risk plaque; BMI, body mass index; TG, triglycerides; LDL-C, low-density lipoprotein cholesterol; Log NT-proBNP, Log N-terminal brain natriuretic peptide precursor.

LASSO regression was used (Figures 4A,B) to identify five variables that affect plaque stability: BMI ≥ 25 kg/m^2^, LHR, TC, TG, and Log NT-proBNP. Combined with the variables screened by LASSO regression and clinically meaningful variables, the above 11 variables were included in a multivariate logistic stepwise regression model to create the HRP risk factor model (Table 3). The final model includes five variables: BMI ≥ 25 kg/m^2^, age, TG, LDL-C, and Log NT-proBNP. The nomogram is constructed (Figure 5). Each predictor is represented by a scale on the left, with the corresponding points derived from the regression coefficients. The total points, calculated by summing the individual points for each predictor, are mapped to the predicted probability of the event occurring on the rightmost scale. The nomogram uses odds ratios for each variable (shown as line markers), indicating how changes in each predictor affect the odds of the outcome. The colored density plot beneath the scales represents the distribution of data for each variable. Logistic regression was used to derive the model, with statistical significance indicated for variables with *P*-values less than 0.05. Confidence intervals for the predicted probabilities are also shown for clarity. A sum score was calculated as the total of the scores for related predictors with the risk of HRP on the basal axis. For example, in a patient with BMI < 25 kg/m^2^, age 74, TG 1.83 mmol/L, LDL-C 4.08 mmol/L and Log NT-proBNP 3.19, the total points was 150, and the 0dds was 1.84.

**Table 3:** Univariate and multivariate logistic regression analysis.

Variables	Univariate logistic regression		Multivariate logistic regression	
	OR（95% CI)	*P* value	OR（95% CI)	*P* value
Male	0.65 (0.28–1.52)	0.324		
Age	1.01 (0.99–1.04)	0.406	1.03 (1.00–1.06)	0.049
Hypertension	0.97 (0.51–1.86)	0.936		
Diabetes	1.84 (0.92–3.7)	0.087		
Smoke	0.84 (0.44–1.6)	0.599		
BMI ≥ 25 kg/m^2^	2.58 (1.33–4.99)	0.005	2.36 (1.12–4.98)	0.025
TC, mmol/L	1.84 (1.31–2.59)	<0.001		
TG, mmol/L	1.57 (1.22–2.02	<0.001	1.58 (1.20–1.07)	0.001
LDL-C, mmol/L	1.80 (1.23–2.62)	0.002	1.87 (1.22–2.86)	0.004
LHR	1.82 (1.31–2.54)	<0.001		
Log NT-proBNP, pg/ml	2.18 (1.24–3.84)	0.007	2.04 (1.12–3.72)	0.021

BMI, body mass index; TC, total cholesterol; TG, triglycerides; LDL-C, low-density lipoprotein cholesterol; LHR, low-density lipoprotein cholesterol to high-density lipoprotein cholesterol ratio; NT-proBNP, N-terminal pro-B-type natriuretic peptide.

The results of the multivariate logistic analysis revealed that the variables listed above are independent risk factors for high risk of HRP (Figure 4C).

**Figure 6:**
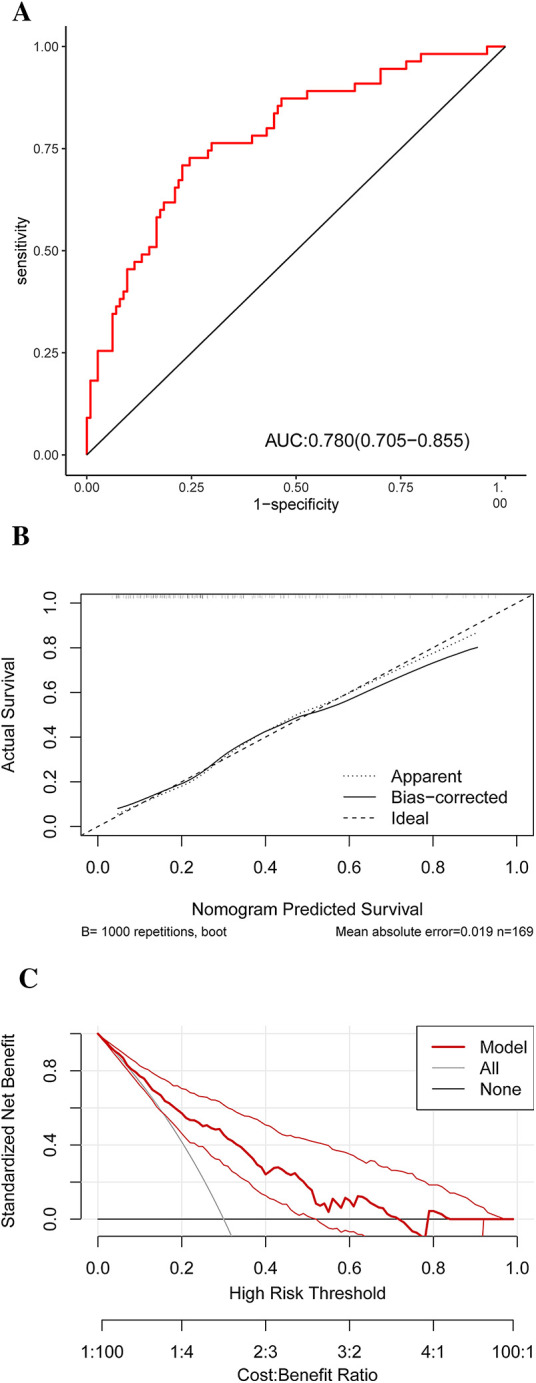
ROC **(A)** result for the diagnostic performances of the HRP risk factor model. A calibration curve **(B)** of the nomogram for probability prediction. The *x*-axis represents the nomogram-predicted probability, while the *y*-axis represents the actual probability. DCA **(C)** demonstrates the net benefit of the HRP risk factor model compared with the strategies of “treating all” or “treating none” for different decision thresholds. ROC, receiver operating characteristic curve analysis; HRP, high-risk plaque; DCA, decision curve analysis.

The discriminatory power of the nomogram was assessed using the area under the ROC curve. The ROC curve analysis revealed that the model had a high predictive capability for HRP, with an AUC of 0.780 [95% confidence interval (CI): 0.705–0.855] (Figure 6A). Internal validation is carried out using bootstrap resampling with a sample size of 1,000, and the calibration curve is plotted. The calibration curves of the model show that the predicted probabilities closely match the actual probabilities (Figure 6B). The DCA was used to assess clinical practicability. The DCA of the model is higher than the reference line, indicating that its clinical utility is generally superior (Figure 6C).

## Discussion

This study successfully developed and validated a nomogram for accurately predicting HRPs in patients with ACS. The nomogram included age, BMI ≥ 25 kg/m^2^, TG, LDL-C, and Log NT-proBNP levels. It demonstrated good discrimination, calibration, and clinical validity, making it a useful and clinically relevant tool for identifying HRPs defined by OCT and ACS.

ACS is typically caused by the rupture of vulnerable plaques, leading to thrombosis (14–16). Previous research has shown that vulnerable plaques have the following characteristics: TCFA, rich in macrophages, and a large central necrotic core (17, 18). The CLIMA study introduced the concept of HRP and found that HRPs are associated with a higher risk of major coronary events (5). Previous research has also found that HRPs defined by OCT are associated with an increased risk of cardiovascular events (6–8). Recent large-scale studies further support these findings: Matsumura et al. showed that OCT-defined TCFA and a minimal lumen area <3.5 mm^2^ are independent predictors of major adverse cardiovascular events (MACE) in patients with acute myocardial infarction (8), while van Veelen et al. reported that HRPs in nonculprit lesions, even when fractional flow reserve (FFR) negative, are linked to a higher incidence of MACE over 2 years (19). These accumulating data reinforce the clinical importance of early HRP detection and timely intervention. However, current imaging modalities, including OCT and intravascular ultrasonography, are invasive, require specialized equipment, and have limitations in the early detection of plaque stability. In low-middle-income countries and areas, OCT is not feasible due to economic and workforce constraints. Circulating biomarkers can be used as additional tools for predicting the risk of vulnerable plaques, and developing a risk prediction model based on circulating biomarkers has greater utility and potential for widespread use in clinical practice. Our proposed biomarker-based model is intended not to replace OCT but to provide a practical, non-invasive tool for early risk stratification and guiding the need for further invasive assessment.

Due to the crucial role of lipid accumulation and inflammation in atherosclerotic plaque formation, previous research on markers of vulnerable plaque circulation has primarily focused on these processes (20). Mechanistically, elevated levels of specific lipids or inflammatory factors indicate their involvement in initiating harmful events that lead to plaque destabilization, increasing the risk of unstable plaque formation. Conversely, certain molecules released by unstable plaque lesions into the bloodstream are easily detectable and may serve as predictors of vulnerable plaques. Hence, the use of circulating biomarkers has a high potential for detecting plaque vulnerability in patients. Nonetheless, no single reliable biological marker has demonstrated adequate sensitivity and specificity. This emphasizes the importance of identifying a panel of circulating markers for predicting the risk of AS plaque vulnerability, as well as developing a risk factor analysis model with greater utility in clinical practice. The current study analyzed relevant circulating blood indicators in patients, revealing statistically significant differences (*P* < 0.05) in BMI, Hs-CRP, Hemoglobin A1c, TG, TC, HDL-C, LDL-C, ApoB/ApoA1 ratio, and NT-proBNP levels between high-risk and non-HRP groups. These commonly used laboratory indicators may act as risk factors for HRPs.

In this study, general clinical data and laboratory examination indicators of patients were incorporated into the HRP risk prediction system. The aforementioned variables were used as influencing factors to identify factors affecting plaque stability using the LASSO regression model, which resulted in the creation of a model. Variables included “age”, “BMI ≥ 25 kg/m^2^”, “TG”, “LDL-C,” and “Log NT-proBNP”. Given the large number of indicators used in this study, there is a high risk of encountering issues such as variable collinearity when selecting variables using the least squares method. This can lead to important variables being overlooked, resulting in inaccuracies in the prediction model. The LASSO regression model addresses these concerns by efficiently selecting variables by compressing regression coefficients to zero (21). In the model development, we used the LASSO–Cox method to estimate the relationship between predictors and HRPs. LASSO regularization is a method for managing overfitting and variable selection that has been widely used in a variety of machine learning algorithms (22). When the LASSO method is applied to the Cox model, the estimation variance is reduced, and a subset of predictors is chosen, resulting in an interpretable Cox model (23). To ensure that the model was accurate, we used a nomogram to simplify the parameters in the model presentation. Furthermore, the nomogram model created using identified HRP risk factors is a simple and intuitive tool. Clinicians can calculate the cumulative scores of risk factors using the nomogram, allowing for quick and easy risk stratification for plaque vulnerability and HRP possibility. Thus, the risk factors evaluated in this study can be easily obtained through patient history collection and routine laboratory examination, allowing for early detection of HRPs. The risk prevention model's AUC of 0.780 (95% CI: 0.705–0.855) developed in this study indicates that assessing HRPs has a high predictive capability.

The results from screening variables in the LASSO regression model found that age, BMI ≥ 25 kg/m^2^, TG, LDL-C, and Log NT-proBNP levels are reliable indicators for predicting HRPs, implying that HRPs can be clinically identified using these indicators alone. Age is well-known as a traditional risk factor for cardiovascular events. Many studies have considered age to be an independent predictor of ACS (24), and in studies focusing on other risk factors, age is typically adjusted (25, 26). Furthermore, BMI ≥ 25 kg/m^2^ was associated with HRP. BMI > 25 kg/m^2^ was recently found to be associated with a signiﬁcantly increased long-term risk of cardiovascular disease morbidity and mortality (27), and another study linked this epidemiologic evidence to HRP formation (28). Elevated triglyceride levels and triglyceride-rich lipoproteins (TRLs) have been increasingly recognized as important contributors to atherosclerotic cardiovascular disease beyond LDL-C (29). Recent evidence suggests that TRLs may promote the formation of lipid-rich necrotic cores, inflammation, and endothelial dysfunction—features commonly associated with high-risk plaques (HRPs) (30). For instance, the accumulation of TRLs in the arterial wall has been shown to induce macrophage activation and foam cell formation, both of which are implicated in plaque vulnerability. These pathophysiological mechanisms highlight the potential role of elevated TG in the development of OCT-defined HRPs and support its inclusion as a relevant biomarker in risk stratification models. LDL-C has long been recognized as an important risk factor for ASCVD, and numerous studies have consistently shown that LDL-C lowering interventions can effectively reduce plaque vulnerability, regardless of the imaging modality used to assess plaque characteristics (31–33). A recent study on intracoronary imaging using OCT found that high levels of small dense LDL-C are linked to the presence of vulnerable plaques (34). NT-proBNP concentration is regarded as a marker of cardiac function in heart disease, and myocardial ischemia can cause a reversible increase in regional wall stress, potentially leading to increased natriuretic peptide release (35). A previous study found significant associations between NT-proBNP and coronary atherosclerotic plaque parameters, which were consistent with the high-sensitivity cardiac Troponin T results (36).

Although HbA1c and diabetes are recognized cardiovascular risk factors, neither was retained in the final model. HbA1c was excluded during the LASSO regression due to its limited independent predictive value after penalization. Diabetes was initially selected but subsequently removed in the multivariate logistic regression, likely due to collinearity with other glycemic markers and limited statistical significance (*P* = 0.08 in univariate analysis). In contrast, variables such as age, overweight BMI, TG, LDL-C, and NT-proBNP showed stronger and more consistent associations with high-risk plaques and were prioritized in the final model. This reflects the relative predictive contributions of different variables within our cohort. Importantly, we acknowledge that the exclusion of glycemic variables may also reflect the limited sample size of our study, which could reduce the statistical power to detect their independent associations. Future large-scale, multicenter investigations are warranted to validate these findings and comprehensively clarify the prognostic value of glycemic factors in cardiovascular risk prediction.

The predictive value of circulating biomarkers for high-risk coronary plaque features has been increasingly supported by evidence from non-invasive imaging studies. Russo et al. demonstrated that low HDL-C and elevated levels of leptin and interleukin-6 were independently associated with high-risk coronary anatomy as assessed by coronary CT angiography (CCTA) in patients with stable chest pain (37). Similarly, Nidorf et al. reported that high-sensitivity C-reactive protein (hsCRP) levels were significantly associated with vulnerable plaque features, including low attenuation and positive remodeling, identified by CCTA (38). These findings underscore the role of systemic inflammation and lipid metabolism in plaque vulnerability and support the integration of serological biomarkers into non-invasive risk prediction models. Our current study aligns with this direction, aiming to develop a practical, blood-based tool for identifying high-risk plaques defined by OCT, especially in settings where intracoronary imaging may not be routinely feasible.

It is important to note that the model's strong predictive performance does not imply that other indicators have a weak predictive effect on HRPs. Previous research has shown that individual indicators are closely related to the onset and progression of HRPs, as exemplified by the European Society of Cardiology/European Atherosclerosis Blood Lipid Management Guidelines, which emphasize the causal relationship between LDL-C and all apolipoprotein B lipoproteins in arteriosclerotic cardiovascular disease. It is suggested that the role of apolipoprotein B and lipoprotein in cardiovascular risk stratification should be investigated further (25). More research is needed to understand the underlying mechanisms of plaque instability, and progression and to develop more reliable biomarkers for the early detection of HRPs in ACS.

Therefore, we believe that our model will help patients better understand the disease and doctors make clinical decisions. Particularly for patients with a high risk of ACS, doctors can use this model to determine whether patients would benefit from treatment.

There are some limitations to our study. First, the single-center design and limited sample size reduced the statistical power, restricted the robustness of subgroup analyses, and lacked external validation, which may affect the generalizability of the results. Second, for safety considerations regarding the use of iodinated contrast, patients with an estimated glomerular filtration rate (eGFR) <60 ml/min/1.73 m^2^ were excluded; Additionally, in a subset of patients (*n* = 26) with severely stenotic or occluded lesions, low-pressure (4–6 atm) balloon pre-dilatation was performed to facilitate OCT catheter passage. These procedures are consistent with standard clinical practice and their impact on plaque parameters (such as MLA and FCT) is minimal or negligible. All images were interpreted by blinded observers, and sensitivity analyses were conducted to minimize potential bias.

Despite these limitations, OCT technology enabled accurate classification of high-risk plaque populations, and rigorous statistical methods were applied for risk prediction, thereby providing the proposed prediction model with high accuracy and reliability.

## Conclusion

In this study, an OCT examination was used to accurately identify HRPs associated with risk factors in the ACS cohort. The nomogram risk prediction model developed in response to these findings has high predictive efficacy and clinical applicability, making it critical for identifying, preventing, and treating HRP vulnerability. However, due to the single-center observational cohort design and limited sample size, future research will focus on validating the nomogram model's clinical utility in multi-center studies with larger samples.

## Data Availability

The datasets generated and/or analyzed during the current study are not publicly available due to patient privacy concerns and institutional regulations. Additionally, the data are part of an ongoing research project and have not been fully released. However, they are available from the corresponding author upon reasonable request and with appropriate institutional approval.
